# (2*E*)-1-(2,5-Dichloro-3-thien­yl)-3-(6-meth­oxy-2-naphth­yl)prop-2-en-1-one

**DOI:** 10.1107/S1600536810022725

**Published:** 2010-06-18

**Authors:** Jerry P. Jasinski, Albert E. Pek, C. S. Chidan Kumar, H. S. Yathirajan, A. N Mayekar

**Affiliations:** a Department of Chemistry, Keene State College, 229 Main Street, Keene, NH 03435-2001, USA; b Department of Studies in Chemistry, University of Mysore, Manasagangotri, Mysore 570 006, India; c SeQuent Scientific Ltd, Baikampady, New Mangalore, 575 011, India

## Abstract

In the title compound, C_18_H_12_Cl_2_O_2_S, the dihedral angle between the thio­phene ring and the naphthalene ring system is 2.13 (4)°. In the crystal, pairs of weak inter­molecular C—H⋯O hydrogen bonds form centrosymmetric dimers.

## Related literature

For the biological activity of thio­phene-containing compounds, see: Ferreira *et al.* (2006 ▶); Bonini *et al.* (2005 ▶); Kulikova *et al.* (1980 ▶). For the anti­radiation activity of thio­phenes, see: Hassan *et al.* (1998 ▶). For the synthesis and anti­microbial evaluation of new chalcones, see: Tomar *et al.* (2007 ▶). For the biological activity of chalcone derivatives, see: Nowakowska *et al.* (2007 ▶). For related structures, see: Butcher *et al.* (2007 ▶); Harrison *et al.* (2007*a* ▶,*b* ▶); Li *et al.* (2009 ▶); Yathirajan *et al.* (2006 ▶). 
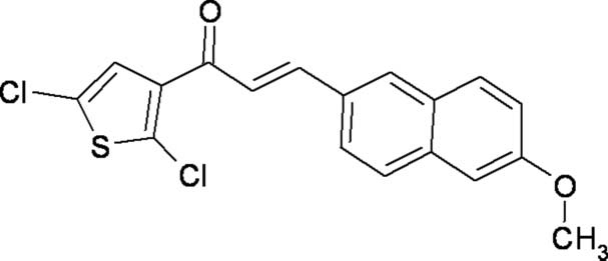


## Experimental

### 

#### Crystal data

C_18_H_12_Cl_2_O_2_S
*M* *_r_* = 363.24
Monoclinic, 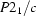

*a* = 7.3237 (5) Å
*b* = 9.4919 (6) Å
*c* = 22.4037 (15) Å
β = 96.183 (1)°
*V* = 1548.35 (18) Å^3^
*Z* = 4
Mo *K*α radiation
μ = 0.56 mm^−1^
*T* = 100 K
0.55 × 0.40 × 0.39 mm

#### Data collection

Bruker APEXII CCD diffractometer
Absorption correction: multi-scan (*SADABS*; Bruker, 2008 ▶) *T*_min_ = 0.748, *T*_max_ = 0.811
17429 measured reflections
4780 independent reflections
4373 reflections with *I* > 2σ(*I*)
*R*_int_ = 0.021

#### Refinement

*R*[*F* ^2^ > 2σ(*F* ^2^)] = 0.030
*wR*(*F* ^2^) = 0.077
*S* = 0.97
4780 reflections
209 parameters
H-atom parameters constrained
Δρ_max_ = 0.51 e Å^−3^
Δρ_min_ = −0.26 e Å^−3^

### 

Data collection: *APEX2* (Bruker, 2008 ▶); cell refinement: *SAINT* (Bruker, 2008 ▶); data reduction: *SAINT*; program(s) used to solve structure: *SHELXTL* (Sheldrick, 2008 ▶); program(s) used to refine structure: *SHELXTL*; molecular graphics: *SHELXTL*; software used to prepare material for publication: *SHELXTL* and *PLATON* (Spek, 2009 ▶).

## Supplementary Material

Crystal structure: contains datablocks global, I. DOI: 10.1107/S1600536810022725/ci5100sup1.cif

Structure factors: contains datablocks I. DOI: 10.1107/S1600536810022725/ci5100Isup2.hkl

Additional supplementary materials:  crystallographic information; 3D view; checkCIF report

## Figures and Tables

**Table 1:** Hydrogen-bond geometry (Å, °)

*D*—H⋯*A*	*D*—H	H⋯*A*	*D*⋯*A*	*D*—H⋯*A*
C2—H18⋯O2^i^	0.93	2.56	3.2051 (14)	127

Symmetry code: (i) 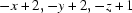.

**Fig. 1.**
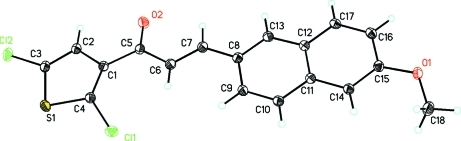
Molecular structure of the title compound, showing the atom labeling scheme and 50% probability displacement ellipsoids.

**Fig. 2.**
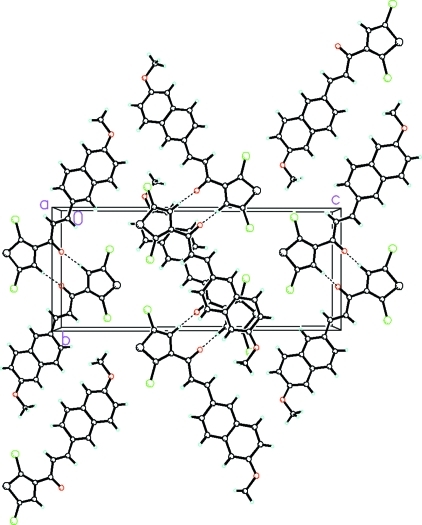
Packing diagram of the title compound, viewed down the a axis. Dashed lines indicate weak C—H···O intermolecular interactions which form dimers.

**Fig. 3.**
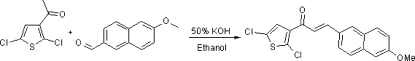
Reaction scheme for the title compound.

## supplementary crystallographic information

### Comment

Thiophenes are important heterocyclic compounds that are widely used as building blocks in many agrochemicals and pharmaceuticals. Thiophene containing compounds are well known to exhibit various biological activities such as antioxidant activity (Ferreira *et al.*, 2006), anti-inflammatory agents and anti-HIV PR inhibitors (Bonini *et al.*, 2005). Thiophene derivatives not only being biologically active (Kulikova *et al.*, 1980), but also show antiradiation activity (Hassan *et al.*, 1998).

The synthesis and antimicrobial evaluation of new chalcones containing 2,5-dichlorothiophene moiety is reported (Tomar *et al.*, 2007). Chalcones have been reported to possess many useful properties, including anti-inflammatory, antimicrobial, antifungal, antioxidant, cytotoxic, antitumor and anticancer activities (Nowakowska *et al.*, 2007). In view of the importance of thiophenes, we report here the crystal structure of the title compound.

In the title molecule, the 2,5-dichloro-3-thienyl and 6-methoxy-2-naphthyl rings are bonded at the opposite ends of the propenone group, the biologically active region (Fig.1). The dihedral angle between mean planes of the dichlorothienyl and naphtyl rings is 2.13 (4)°. The angles between the mean plane of the prop-2-en-1-one group and the mean planes of the thienyl and naphtyl rings are 3.08 (4)°, and 2.88 (4)° repectively. In the crystal, pairs of weak intermolecular C2—H18···O2 hydrogen bonds form dimers and contribute to crystal stability.

### Experimental

1-(2,5-Dichlorothiophen-3-yl)ethanone (1.95 g, 0.01 mol) was mixed with 6-methoxy-2-naphthaldehyde (1.86 g, 0.01 mol) and dissolved in ethanol (30 ml). To this, 3 ml of KOH (50%) was added (Fig. 3). The reaction mixture was stirred for 6 h. The resulting crude solid was filtered, washed successively with distilled water and finally recrystallized from ethanol (95%) to give the pure chalcone. Single crystals suitable for *X*-ray diffraction studies were grown by the slow evaporation of the acetone-toluene (1:1) solution (m.p. 401–403 K).

### Refinement

H atoms were placed in their calculated positions and then refined using the riding model with C–H = 0.93 or 0.96 Å, and with *U*_iso_(H) = 1.18–1.50*U*_eq_(C).

### Crystal data

**Table:**

C_18_H_12_Cl_2_O_2_S	*F*(000) = 744
*M**_r_* = 363.24	*D*_x_ = 1.558 Mg m^−^^3^
Monoclinic, *P*2_1_/*c*	Mo *K*α radiation, λ = 0.71073 Å
Hall symbol: -P 2ybc	Cell parameters from 9959 reflections
*a* = 7.3237 (5) Å	θ = 2.3–31.3°
*b* = 9.4919 (6) Å	µ = 0.56 mm^−^^1^
*c* = 22.4037 (15) Å	*T* = 100 K
β = 96.183 (1)°	Block, yellow
*V* = 1548.35 (18) Å^3^	0.55 × 0.40 × 0.39 mm
*Z* = 4	

### Data collection

**Table:**

Bruker APEXII CCD diffractometer	4780 independent reflections
Radiation source: fine-focus sealed tube	4373 reflections with *I* > 2σ(*I*)
graphite	*R*_int_ = 0.021
ω scans	θ_max_ = 31.4°, θ_min_ = 1.8°
Absorption correction: multi-scan (*SADABS*; Bruker, 2008)	*h* = −10→10
*T*_min_ = 0.748, *T*_max_ = 0.811	*k* = −13→13
17429 measured reflections	*l* = −31→31

### Refinement

**Table:**

Refinement on *F*^2^	Primary atom site location: structure-invariant direct methods
Least-squares matrix: full	Secondary atom site location: difference Fourier map
*R*[*F*^2^ > 2σ(*F*^2^)] = 0.030	Hydrogen site location: inferred from neighbouring sites
*wR*(*F*^2^) = 0.077	H-atom parameters constrained
*S* = 0.97	*w* = 1/[σ^2^(*F*_o_^2^) + (0.0374*P*)^2^ + 1.0425*P*] where *P* = (*F*_o_^2^ + 2*F*_c_^2^)/3
4780 reflections	(Δ/σ)_max_ = 0.001
209 parameters	Δρ_max_ = 0.51 e Å^−^^3^
0 restraints	Δρ_min_ = −0.26 e Å^−^^3^

### Special details

**Table:**

Geometry. All e.s.d.'s (except the e.s.d. in the dihedral angle between two l.s. planes) are estimated using the full covariance matrix. The cell e.s.d.'s are taken into account individually in the estimation of e.s.d.'s in distances, angles and torsion angles; correlations between e.s.d.'s in cell parameters are only used when they are defined by crystal symmetry. An approximate (isotropic) treatment of cell e.s.d.'s is used for estimating e.s.d.'s involving l.s. planes.
Refinement. Refinement of *F*^2^ against ALL reflections. The weighted *R*-factor *wR* and goodness of fit *S* are based on *F*^2^, conventional *R*-factors *R* are based on *F*, with *F* set to zero for negative *F*^2^. The threshold expression of *F*^2^ > σ(*F*^2^) is used only for calculating *R*-factors(gt) *etc*. and is not relevant to the choice of reflections for refinement. *R*-factors based on *F*^2^ are statistically about twice as large as those based on *F*, and *R*- factors based on ALL data will be even larger.

### Fractional atomic coordinates and isotropic or equivalent isotropic displacement parameters (Å^2^)

**Table:**

	*x*	*y*	*z*	*U*_iso_*/*U*_eq_	
Cl1	0.97320 (4)	0.57727 (3)	0.666046 (12)	0.02001 (7)	
Cl2	1.12452 (4)	1.16879 (3)	0.677155 (14)	0.02261 (7)	
S1	1.08482 (4)	0.86262 (3)	0.702059 (12)	0.01784 (7)	
O1	0.35474 (12)	−0.11321 (9)	0.30656 (4)	0.01761 (16)	
O2	0.81055 (13)	0.85374 (9)	0.49460 (4)	0.02136 (18)	
C1	0.93801 (15)	0.81919 (11)	0.59314 (5)	0.01381 (19)	
C2	0.97881 (15)	0.96709 (12)	0.59807 (5)	0.01517 (19)	
H18	0.9548	1.0307	0.5667	0.018*	
C3	1.05603 (15)	1.00339 (12)	0.65347 (5)	0.0166 (2)	
C4	0.99005 (15)	0.75105 (12)	0.64645 (5)	0.01516 (19)	
C5	0.84960 (15)	0.76639 (12)	0.53422 (5)	0.01448 (19)	
C6	0.81083 (16)	0.61554 (12)	0.52385 (5)	0.0159 (2)	
H13	0.8391	0.5501	0.5544	0.019*	
C7	0.73400 (15)	0.57431 (12)	0.46967 (5)	0.0159 (2)	
H12	0.7128	0.6448	0.4409	0.019*	
C8	0.67971 (15)	0.43260 (11)	0.45033 (5)	0.01457 (19)	
C9	0.70770 (15)	0.31296 (12)	0.48855 (5)	0.01504 (19)	
H9	0.7650	0.3241	0.5274	0.018*	
C10	0.65144 (15)	0.18169 (12)	0.46900 (5)	0.01494 (19)	
H8	0.6709	0.1050	0.4947	0.018*	
C11	0.56360 (14)	0.16097 (11)	0.40986 (5)	0.01316 (19)	
C12	0.53666 (14)	0.27935 (11)	0.37112 (5)	0.01365 (19)	
C13	0.59605 (15)	0.41327 (11)	0.39266 (5)	0.0152 (2)	
H11	0.5783	0.4907	0.3673	0.018*	
C14	0.50474 (15)	0.02566 (11)	0.38918 (5)	0.01431 (19)	
H2	0.5229	−0.0522	0.4143	0.017*	
C15	0.42044 (15)	0.01046 (12)	0.33156 (5)	0.01419 (19)	
C16	0.39546 (15)	0.12772 (12)	0.29236 (5)	0.0157 (2)	
H6	0.3402	0.1154	0.2533	0.019*	
C17	0.45213 (15)	0.25871 (12)	0.31159 (5)	0.0155 (2)	
H5	0.4353	0.3350	0.2855	0.019*	
C18	0.37100 (18)	−0.23386 (13)	0.34421 (6)	0.0225 (2)	
H1A	0.4978	−0.2487	0.3586	0.034*	
H1B	0.3246	−0.3149	0.3218	0.034*	
H1C	0.3016	−0.2196	0.3777	0.034*	

### Atomic displacement parameters (Å^2^)

**Table:**

	*U*^11^	*U*^22^	*U*^33^	*U*^12^	*U*^13^	*U*^23^
Cl1	0.02618 (14)	0.01646 (13)	0.01724 (13)	0.00090 (10)	0.00169 (10)	0.00379 (9)
Cl2	0.02353 (14)	0.01989 (14)	0.02423 (14)	−0.00595 (10)	0.00179 (10)	−0.00808 (10)
S1	0.01786 (13)	0.02130 (14)	0.01387 (12)	−0.00047 (10)	−0.00050 (9)	−0.00146 (10)
O1	0.0221 (4)	0.0145 (4)	0.0154 (4)	−0.0021 (3)	−0.0015 (3)	−0.0017 (3)
O2	0.0305 (5)	0.0157 (4)	0.0167 (4)	−0.0021 (3)	−0.0032 (3)	0.0020 (3)
C1	0.0133 (4)	0.0139 (4)	0.0142 (4)	−0.0005 (3)	0.0019 (3)	−0.0009 (4)
C2	0.0150 (5)	0.0142 (5)	0.0163 (5)	−0.0010 (4)	0.0016 (4)	−0.0012 (4)
C3	0.0154 (5)	0.0164 (5)	0.0181 (5)	−0.0025 (4)	0.0022 (4)	−0.0029 (4)
C4	0.0150 (5)	0.0154 (5)	0.0151 (5)	−0.0006 (4)	0.0016 (4)	−0.0007 (4)
C5	0.0143 (5)	0.0147 (5)	0.0145 (4)	−0.0014 (4)	0.0017 (3)	−0.0010 (4)
C6	0.0189 (5)	0.0129 (5)	0.0156 (5)	−0.0005 (4)	0.0009 (4)	0.0005 (4)
C7	0.0173 (5)	0.0141 (5)	0.0161 (5)	−0.0003 (4)	0.0015 (4)	0.0000 (4)
C8	0.0145 (5)	0.0139 (5)	0.0154 (5)	−0.0005 (4)	0.0020 (4)	−0.0006 (4)
C9	0.0163 (5)	0.0156 (5)	0.0128 (4)	−0.0007 (4)	−0.0004 (4)	−0.0003 (4)
C10	0.0164 (5)	0.0146 (5)	0.0134 (4)	0.0003 (4)	−0.0004 (4)	0.0016 (4)
C11	0.0122 (4)	0.0142 (5)	0.0131 (4)	0.0002 (3)	0.0013 (3)	−0.0002 (3)
C12	0.0127 (4)	0.0148 (5)	0.0135 (4)	0.0008 (4)	0.0012 (3)	0.0000 (4)
C13	0.0170 (5)	0.0135 (5)	0.0148 (5)	0.0001 (4)	0.0010 (4)	0.0014 (4)
C14	0.0154 (5)	0.0138 (5)	0.0134 (4)	0.0000 (4)	0.0003 (3)	0.0004 (4)
C15	0.0131 (4)	0.0147 (5)	0.0148 (5)	−0.0003 (4)	0.0018 (3)	−0.0019 (4)
C16	0.0152 (5)	0.0187 (5)	0.0130 (4)	0.0007 (4)	0.0000 (4)	−0.0006 (4)
C17	0.0166 (5)	0.0170 (5)	0.0126 (4)	0.0007 (4)	0.0004 (4)	0.0016 (4)
C18	0.0285 (6)	0.0152 (5)	0.0223 (6)	−0.0033 (4)	−0.0042 (5)	0.0007 (4)

### Geometric parameters (Å, °)

**Table:**

Cl1—C4	1.7148 (12)	C9—C10	1.3694 (15)
Cl2—C3	1.7148 (12)	C9—H9	0.93
S1—C3	1.7220 (12)	C10—C11	1.4230 (15)
S1—C4	1.7231 (11)	C10—H8	0.93
O1—C15	1.3657 (13)	C11—C14	1.4167 (15)
O1—C18	1.4198 (14)	C11—C12	1.4206 (15)
O2—C5	1.2257 (14)	C12—C13	1.4116 (15)
C1—C4	1.3756 (15)	C12—C17	1.4220 (15)
C1—C2	1.4371 (15)	C13—H11	0.93
C1—C5	1.4930 (15)	C14—C15	1.3772 (15)
C2—C3	1.3519 (15)	C14—H2	0.93
C2—H18	0.93	C15—C16	1.4174 (15)
C5—C6	1.4732 (15)	C16—C17	1.3658 (16)
C6—C7	1.3403 (15)	C16—H6	0.93
C6—H13	0.93	C17—H5	0.93
C7—C8	1.4553 (15)	C18—H1A	0.96
C7—H12	0.93	C18—H1B	0.96
C8—C13	1.3811 (15)	C18—H1C	0.96
C8—C9	1.4238 (15)		
			
C3—S1—C4	90.43 (5)	C9—C10—H8	119.5
C15—O1—C18	116.50 (9)	C11—C10—H8	119.5
C4—C1—C2	110.89 (10)	C14—C11—C12	120.00 (10)
C4—C1—C5	131.54 (10)	C14—C11—C10	121.40 (10)
C2—C1—C5	117.57 (10)	C12—C11—C10	118.60 (10)
C3—C2—C1	112.49 (10)	C13—C12—C11	119.07 (10)
C3—C2—H18	123.8	C13—C12—C17	122.12 (10)
C1—C2—H18	123.8	C11—C12—C17	118.80 (10)
C2—C3—Cl2	126.65 (9)	C8—C13—C12	121.93 (10)
C2—C3—S1	113.15 (9)	C8—C13—H11	119.0
Cl2—C3—S1	120.20 (7)	C12—C13—H11	119.0
C1—C4—Cl1	130.82 (9)	C15—C14—C11	119.43 (10)
C1—C4—S1	113.03 (8)	C15—C14—H2	120.3
Cl1—C4—S1	116.14 (6)	C11—C14—H2	120.3
O2—C5—C6	121.16 (10)	O1—C15—C14	125.24 (10)
O2—C5—C1	117.28 (10)	O1—C15—C16	113.86 (9)
C6—C5—C1	121.57 (10)	C14—C15—C16	120.89 (10)
C7—C6—C5	118.79 (10)	C17—C16—C15	120.32 (10)
C7—C6—H13	120.6	C17—C16—H6	119.8
C5—C6—H13	120.6	C15—C16—H6	119.8
C6—C7—C8	127.89 (10)	C16—C17—C12	120.54 (10)
C6—C7—H12	116.1	C16—C17—H5	119.7
C8—C7—H12	116.1	C12—C17—H5	119.7
C13—C8—C9	118.50 (10)	O1—C18—H1A	109.5
C13—C8—C7	118.74 (10)	O1—C18—H1B	109.5
C9—C8—C7	122.76 (10)	H1A—C18—H1B	109.5
C10—C9—C8	120.97 (10)	O1—C18—H1C	109.5
C10—C9—H9	119.5	H1A—C18—H1C	109.5
C8—C9—H9	119.5	H1B—C18—H1C	109.5
C9—C10—C11	120.92 (10)		
			
C4—C1—C2—C3	−0.59 (14)	C8—C9—C10—C11	0.10 (17)
C5—C1—C2—C3	179.12 (10)	C9—C10—C11—C14	−179.96 (10)
C1—C2—C3—Cl2	179.91 (8)	C9—C10—C11—C12	0.54 (16)
C1—C2—C3—S1	0.38 (13)	C14—C11—C12—C13	179.89 (10)
C4—S1—C3—C2	−0.07 (9)	C10—C11—C12—C13	−0.61 (15)
C4—S1—C3—Cl2	−179.63 (8)	C14—C11—C12—C17	−0.70 (16)
C2—C1—C4—Cl1	179.61 (9)	C10—C11—C12—C17	178.80 (10)
C5—C1—C4—Cl1	−0.04 (19)	C9—C8—C13—C12	0.61 (17)
C2—C1—C4—S1	0.53 (12)	C7—C8—C13—C12	−178.84 (10)
C5—C1—C4—S1	−179.12 (10)	C11—C12—C13—C8	0.03 (16)
C3—S1—C4—C1	−0.28 (9)	C17—C12—C13—C8	−179.36 (10)
C3—S1—C4—Cl1	−179.50 (7)	C12—C11—C14—C15	−0.45 (16)
C4—C1—C5—O2	176.66 (12)	C10—C11—C14—C15	−179.94 (10)
C2—C1—C5—O2	−2.97 (15)	C18—O1—C15—C14	1.95 (16)
C4—C1—C5—C6	−3.43 (18)	C18—O1—C15—C16	−178.24 (10)
C2—C1—C5—C6	176.94 (10)	C11—C14—C15—O1	−178.83 (10)
O2—C5—C6—C7	1.01 (17)	C11—C14—C15—C16	1.37 (16)
C1—C5—C6—C7	−178.90 (10)	O1—C15—C16—C17	179.05 (10)
C5—C6—C7—C8	−178.11 (11)	C14—C15—C16—C17	−1.13 (17)
C6—C7—C8—C13	177.68 (12)	C15—C16—C17—C12	−0.06 (17)
C6—C7—C8—C9	−1.74 (19)	C13—C12—C17—C16	−179.65 (10)
C13—C8—C9—C10	−0.68 (17)	C11—C12—C17—C16	0.96 (16)
C7—C8—C9—C10	178.74 (11)		

### Hydrogen-bond geometry (Å, °)

**Table:**

*D*—H···*A*	*D*—H	H···*A*	*D*···*A*	*D*—H···*A*
C2—H18···O2^i^	0.93	2.56	3.2051 (14)	127

Symmetry codes: (i) −*x*+2, −*y*+2, −*z*+1.
